# A global multi catchment and multi dataset synthesis for water fluxes and storage changes on land

**DOI:** 10.1038/s41597-024-04203-1

**Published:** 2024-12-05

**Authors:** Mohanna Zarei, Georgia Destouni

**Affiliations:** 1 Department of Physical Geography, Stockholm University, Stockholm, Sweden; 2 Department of Sustainable Development, Environmental Science and Engineering, KTH Royal Institute of Technology, Stockholm, Sweden; 3 Stellenbosch Institute for Advanced Study, Stellenbosch, South Africa

**Keywords:** Hydrology, Climate sciences

## Abstract

Water on land is essential for all societal, ecosystem, and planetary health aspects and conditions, and all life as we know it. Many disciplines consider and model similar terrestrial water phenomena and processes, but comparisons and consistent validations are lacking for the datasets used by various science communities for different world parts, scales, and applications. Here, we present a new global data synthesis that includes and harmonises four comparative datasets for main terrestrial water fluxes and storage changes, and the catchment-wise water balance closure they imply for the 30-year period 1980–2010 in 1561 non-overlapping hydrological catchments around the world. This can be used to identify essential agreements and disagreements of the comparative datasets for spatial variations and temporal changes of runoff, evapotranspiration, water storage, and associated water-balances around the global land area, e.g., for pattern recognition and hypothesis/model testing. The facilitated direct dataset comparison can advance a more coherent, realistic cross-disciplinary understanding of Earth’s water states and changes across regions and scales, from local and up to continental and global.

## Background & Summary

The flows and storage changes of water on land need to be understood and predicted for various, often partly overlapping, science and practice problems addressed in and across many science disciplines, such as environmental, ecological, biogeochemical, geological, atmospheric, climate, and related engineering sciences, in addition to the more water-focused hydrological and water resource science and engineering fields. How consistently and realistically different disciplines understand and represent the terrestrial water system functioning and its variations and temporal changes around the world, from local to global scale, is largely unknown yet key for coherently answering many essential research questions^1^, e.g., regarding the interplay of water on land with climate^2,3^ and human activities^4–6^.

Various datasets may be used to represent and understand the terrestrial water system in different disciplines, based on different combinations of ground-measured, satellite, and model-based data^7–12^. If the water system representations differ substantially between datasets^13,14^, however, only some can be correct while others are inaccurate and unrealistic, and we need to know which. The balance (continuity) of water fluxes and storage changes is a fundamental key to investigating the terrestrial water system and possible dataset agreements and disagreements about it for different parts of the global land area^6,13,15,16^. Hydrological catchments are natural, topographically determined spatial units for such water balance-based investigation of the conditions and balances of water on land^13–15,17,18^. Any measurement station for water flow (or any point) in a landscape and any shoreline of a river, lake or coastal water body has an associated contributing catchment, over which water balance closure can be expressed as DS = P-ET-R, based on the catchment-average main water fluxes of precipitation (P), evapotranspiration (ET) and runoff (R), and the average water storage change per unit catchment area (DS) that a possible imbalance of these fluxes implies. The total average DS may then include changes in subsurface and surface water storages and levels and, for polar and other cold regions, also in the frozen water storage of glaciers and permafrost.

There is now increasing accessibility to data with worldwide spreading for at least some of these main water flux and storage-change variables from different types of sources. These include purely observational global datasets, for example, for: P from the Global Precipitation Climatology Centre-Version7 (GPCC-V7)^19^; and R from the Global Streamflow Indices and Metadata (GSIM)^20,21^. They also include multiple model-based data, for example, for: ET and the soil moisture (SM) component of total water storage from the Global Land Evaporation Amsterdam Model (GLEAM)^22,23^, which combines satellite data with a set of model algorithms for ET and SM; and P, R, ET, and SM from the Global Land Data Assimilation System (GLDAS)^24,25^ based on global land surface modelling, or from the European Centre for Medium-Range Weather Forecasts (ECMWF) Reanalysis 5th Generation product ERA5^26^ based on global climate modelling.

Purely observational data that fully cover whole catchments are only available for R, from monitoring of stream discharges that integrate the total runoff from the entire associated hydrological catchments, yielding catchment-average R from the measured discharge divided by the contributing catchment area. Relatively many meteorological monitoring stations are further available for measurement-based inter/extrapolation of catchment-average P. Considerable fewer ET flux measurement stations are available for measurement-based inter/extrapolation of catchment-average ET, which therefore is instead commonly modelled. A catchment-average water balance can then be calculated by combining observational data for R and P, e.g., from GSIM^20,21^ and GPCC-V7^19^, respectively, with modelled data for ET, e.g., from GLEAM^22,23^, or from a simpler model, such as ET≈P-R assuming negligible average storage change (DS≈0)^6^. Alternatively, ET = P-R-DS can be estimated from satellite data^27^ or calculated from the ground-measured data for R and P combined with ground and/or satellite data for DS^28,29^, which are not commonly available at consistent spatial coverage or resolution across a wide range of different catchment scales. For fully model-based closure of catchment water balances, the reanalysis products GLDAS^24,25^ and ERA5^26^ provide a complete set of P, ET and R data, from which the associated catchment-average storage change can be calculated as DS = P-ET-R.

Terrestrial water balances can thus be determined for catchments of various scales and locations based on different combinations of measured and modelled data, which cannot - without actual comparative testing - be just assumed to consistently and realistically reveal the variations and changes of the terrestrial water conditions around the world. If different dataset representations of the water fluxes, storage changes, and related water balances on land differ substantially, only some can possibly be right, while the others are then inaccurate, and we need to know which are which. The water balance implications of different (types of) datasets therefore need to be directly compared to identify key agreements and disagreements and assess the realism of their various data-model combinations.

**Fig. 1:**
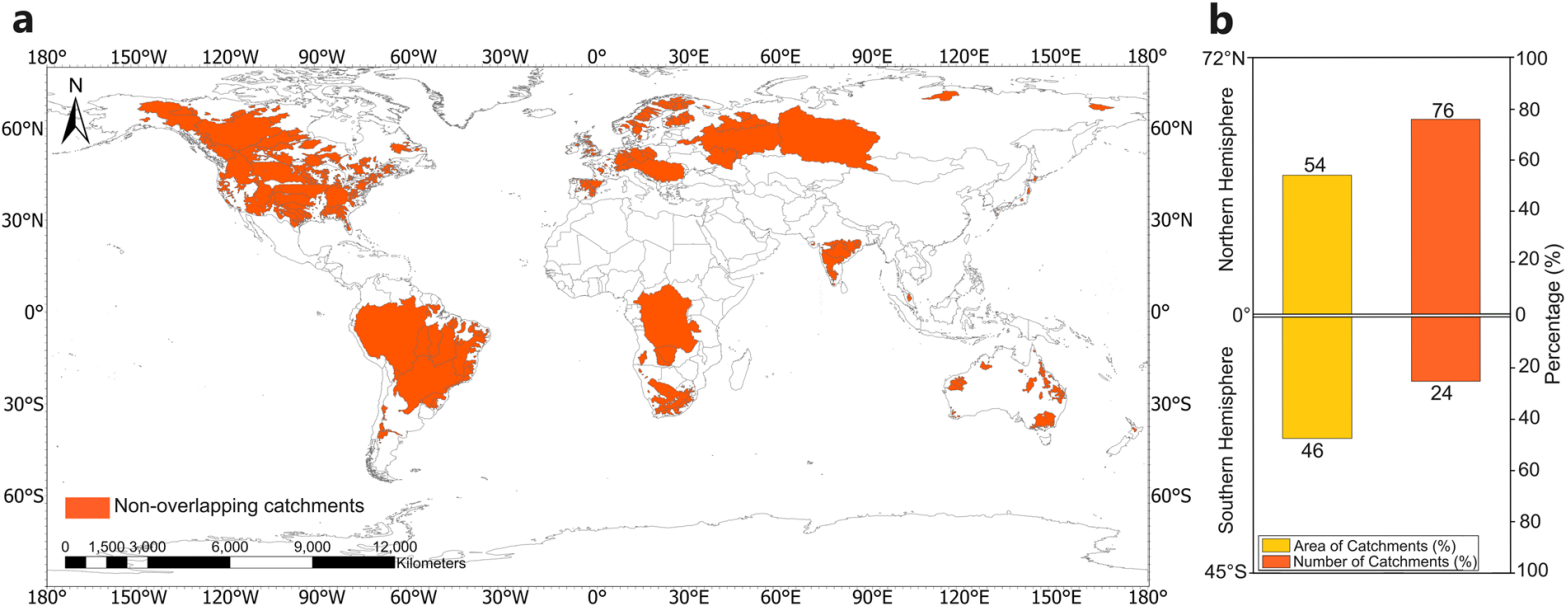
The catchments in the new Global Hydro-Climatic Data (GHCD) synthesis and their spreading around the world. (**a**) Map showing the location of the 1561 non-overlapping catchments (orange fields). (**b**) The number (orange) and the area coverage (yellow) of the catchments in the Northern and the Southern Hemisphere.

To support scientists and practitioners from different disciplines and sectors in such comparative studies and assessments, we here provide a harmonized global data synthesis for 1561 non-overlapping hydrological catchments spread around the world (Fig. 1) based on four comparative datasets with different data-model combinations (Table 1). The dataset “Obs” synthesizes *in-situ* observational data for R^20,21^ and P^19^ and modelled data for the associated average annual ET, based on the simple model ET≈P-R assuming negligible average annual storage change (DS≈0). For direct comparison with this simple ET model and DS assumption, the dataset “Mixed” synthesizes the same observational R^20,21^ and P^19^ data as “Obs” but differs in the model used for ET: “Mixed” uses the more complex global ET model GLEAM^22,23^, based on which the associated storage change can be calculated as DS = P-ET-R. For further comparison of the terrestrial water balance implications of different types of global modelling, the datasets “GLDAS” and “ERA5” are also included with each providing a complete set of model data for P, ET and R, from which the associated DS = P-ET-R can be calculated based fully on the global reanalysis products GLDAS^24,25^ and ERA5^26^, respectively.

**Table 1:** The four comparative datasets in the new multi catchment data synthesis.

	“Obs”	“Mixed”	“GLDAS”	“ERA5”
Precipitation	GPCC-V7^**a**^	GPCC-V7^**a**^	GLDAS NOAH025 M2.0^**f**^	ERA5^**g**^
Evapotranspiration	Water Balance^**b**^	GLEAM v3.3a^**e**^	GLDAS NOAH025 M2.0^**f**^	ERA5^**g**^
Runoff	GSIM^**c**^	GSIM^**c**^	GLDAS NOAH025 M2.0^**f**^	ERA5^**g**^
Soil moisture	—	GLEAM v3.3a^**e**^	GLDAS NOAH025 M2.0^**f**^	ERA5^**g**^
Temperature	GHCN-CAMS^**d**^	GHCN-CAMS^**d**^	GLDAS NOAH025 M2.0^**f**^	ERA5^**g**^

^a^Global Precipitation Climatology Centre-Version7 (GPCC-V7)^19^.

^b^Modelled as ET≈P-R based on average precipitation P and runoff R, and assumed zero storage change (DS≈0).

^c^Global Streamflow Indices and Metadata (GSIM)^20,21^.

^d^Global Historical Climatology Network-Climate Anomaly Monitoring System (GHCN-CAMS)^30^.

^e^Global Land Evaporation Amsterdam Model (GLEAM)^22,23^.

^f^Global Land Data Assimilation System (GLDAS) NOAH025 M2.0^24^,^25^.

^g^European Centre for Medium-Range Weather Forecasts (ECMWF) Reanalysis 5th Generation (ERA5)^26^.

For additional comparisons also of the climate conditions of average air temperature (T), soil moisture (SM), and temporal change in SM (DSM) - as an important component of total storage change DS - over each catchment, data for T, SM and DSM are also included in the datasets (Table 1). The source for T data^30^ is the same in “Obs” and “Mixed”, so that these datasets still differ only in their ET modelling based on the same other hydro-climatic data. The SM data in “Mixed” are from GLEAM^22,23^, consistently with the ET model data in that dataset. The “Obs” dataset simply assumes DS≈0, which can be compared with the DS and DSM data in the other datasets without need to select some particular SM and DSM data to include in “Obs” itself. The T and SM data in “GLDAS” and “ERA5” are from the GLDAS^24,25^ and ERA5^26^ reanalysis products, respectively, consistently with the other data in each of these datasets.

The comparative datasets include data time series of monthly and annual average variable values, and corresponding long-term average values over the 30-year climatological period 1980–2010; only the “Obs” dataset does not include monthly ET and DS time series because its model of average ET≈P-R and DS≈0 is only physically reasonable on average over at least one whole or more years, not each month. The overall data period 1980–2010 meets the World Meteorological Organization (WMO) recommendation to consider 30 years of data for relevant representation of average climatic conditions^31^. Furthermore, the time series and long-term average values synthesized in each dataset represent catchment-average conditions. For R in “Obs” and “Mixed”, the spatial catchment averaging is obtained directly from the catchment-integrating measured discharge data divided by the contributing catchment area. For P in all datasets, as for ET in “Mixed”, “GLDAS” and “ERA5”, and R in “GLDAS” and “ERA5”, the underlying data are gridded and are here spatially aggregated and averaged catchment-wise. For ET in “Obs”, the catchment averaging is obtained directly from the simple model of average catchment water balance ET≈P-R. The datasets also include: catchment-average T, SM and DSM data; data for a DS-implied calculated cumulative water level change (CWLC); and data for the catchment-characteristic relationships quantified by the long-term average aridity index (PET/P), with PET being the T-dependent potential evapotranspiration, and the flux partitioning indices of long-term average ET/P and R/P; however, “Obs” does not include CWLC data since its DS≈0 assumption also implies CWLC = 0.

**Fig. 2:**
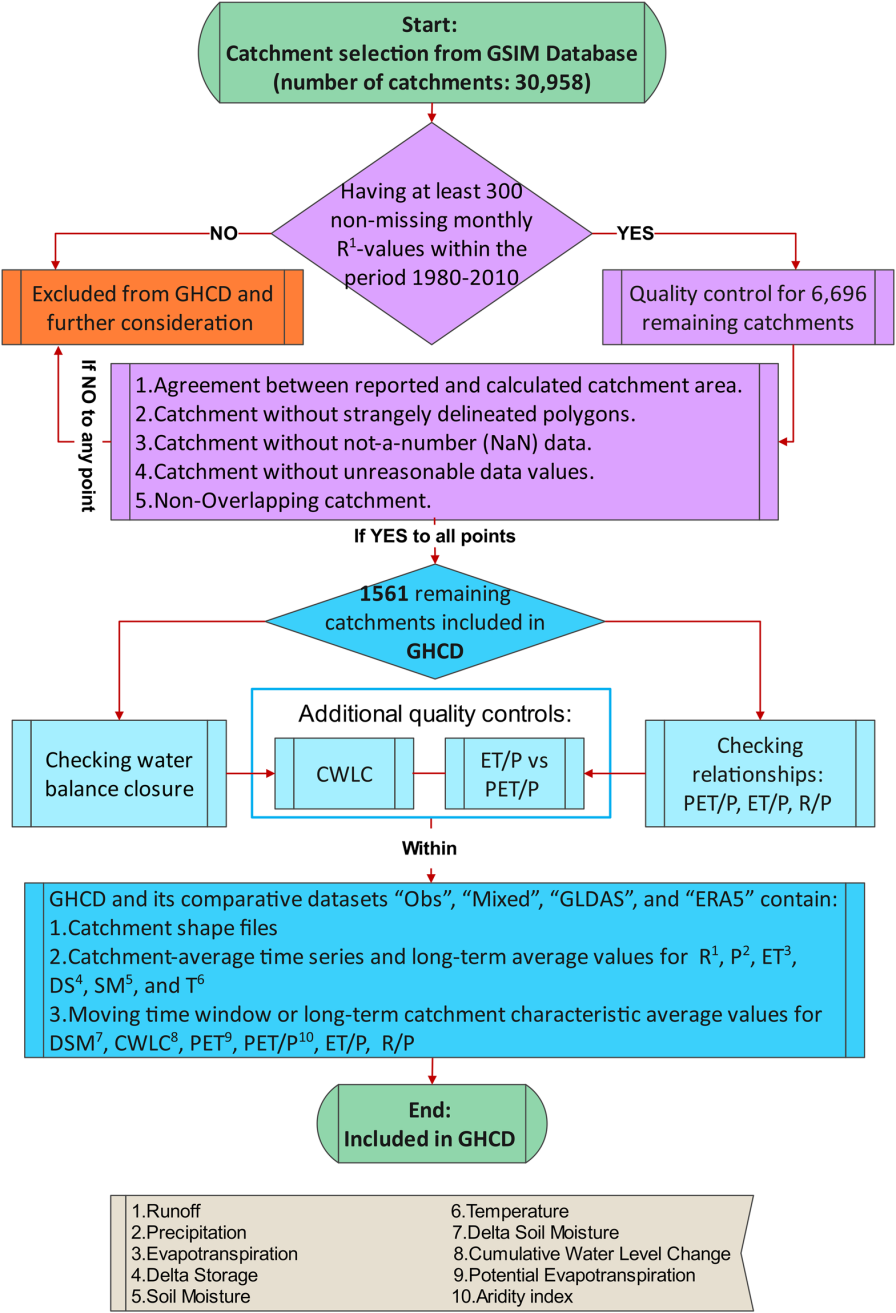
Schematic illustration of the process and steps for creating the new Global Hydro-Climatic Data (GHCD) synthesis.

The schematic flowchart in Fig. 2 illustrates the overall process and main steps for creating the described new multi catchment and multi dataset synthesis, named the Global Hydro-Climatic Data (GHCD)^32^. This can be used to answer dataset-comparative research questions such as: (i) How do different observational and model-based reanalysis datasets close water balances on land within each and across multiple study catchments around the world? (ii) Which water balance outcomes are consistent and which diverge between the different datasets? (iii) Which of the divergent implications are more/less realistic? The data synthesis can also be used to explore many additional research questions in and across various regions and scales. For example, it facilitates assessment of: (iv) spatial variation and temporal change patterns of water conditions around the global land area, and their (v) main drivers and (vi) societal and environmental impacts, e.g., for sectoral water use and ecosystem health. It also facilitates: (vii) calibration and validation of terrestrial water system models of various regions and scales; and (viii) uncertainty quantification and knowledge gap identification associated with divergent dataset outcomes in the above-mentioned and other terrestrial water contexts.

## Methods

### Dataset and catchment selection

Table 1 describes the combinations and sources of data compiled and included in the four comparative datasets of the new multi catchment and multi dataset synthesis GHCD^32^. The GSIM data^20,21^ included in the “Obs” and “Mixed” datasets of GHCD are observational R data time series based on measured water discharges (stream flows) at different hydrometric stations, the locations of which also determine the associated contributing catchments. The other catchment data time series in “Obs” and “Mixed” are based on gridded data products from meteorological observation stations around the world: GPCC-V7^19^ for P and GHCN-CAMS^30^ for T. Furthermore, GHCD includes modelled catchment data for: ET as ET = P-R in “Obs”; ET and SM from GLEAM^22,23^ in “Mixed”; and all included variables from the reanalysis products GLDAS^24,25^ and ERA5^26^ in “GLDAS” and “ERA5”, respectively (Table 1).

Figure 1 shows the locations and area extents of the 1561 non-overlapping hydrological catchments with relevant data for inclusion in GHCD, which cover a total land area of ∼36*10^6^ km^2^. These 1561 catchments were the only ones fulfilling basic criteria of full consistency in spatiotemporal data coverage with open accessibility for direct comparability between the four datasets, while also being non-overlapping and covering the largest total part of the global land area. The criteria were: open access availability of at least 300 non-missing monthly runoff values over the total period 1980–2010 for all four comparative datasets; the parts of the world not included in this new multi catchment and multi dataset synthesis do not fulfil these criteria for all datasets. The non-overlapping criterion was posed so as not to over-represent the same data points in data aggregations and statistics for multiple overlapping catchments. The criterion of largest global land area coverage was fulfilled by including the largest possible non-overlapping catchments that also fulfilled all other basic criteria.

Catchment inclusion was based on the availability of ground observational data for discharge (stream flow) in the “Obs” and “Mixed” datasets. The locations of the discharge measurement stations directly define the contributing hydrological catchments, and determine the associated catchment-average runoff depth R (as measured discharge divided by the contributing catchment area). The data for R are the only available purely observational and fully catchment-integrating data among those required for catchment-wise water balance closure. For direct comparability of catchment-average data across the four datasets (Table 1), we therefore used the availability of observational R data in GSIM (included in “Obs” and “Mixed”) as basis for selecting the catchments included in GHCD. The Technical Validation section describes also additional quality considerations and controls and Fig. 2 shows the whole process and all main quality control steps leading to the final selection of GSIM catchments included in GHCD (Fig. 1).

**Fig. 3:**
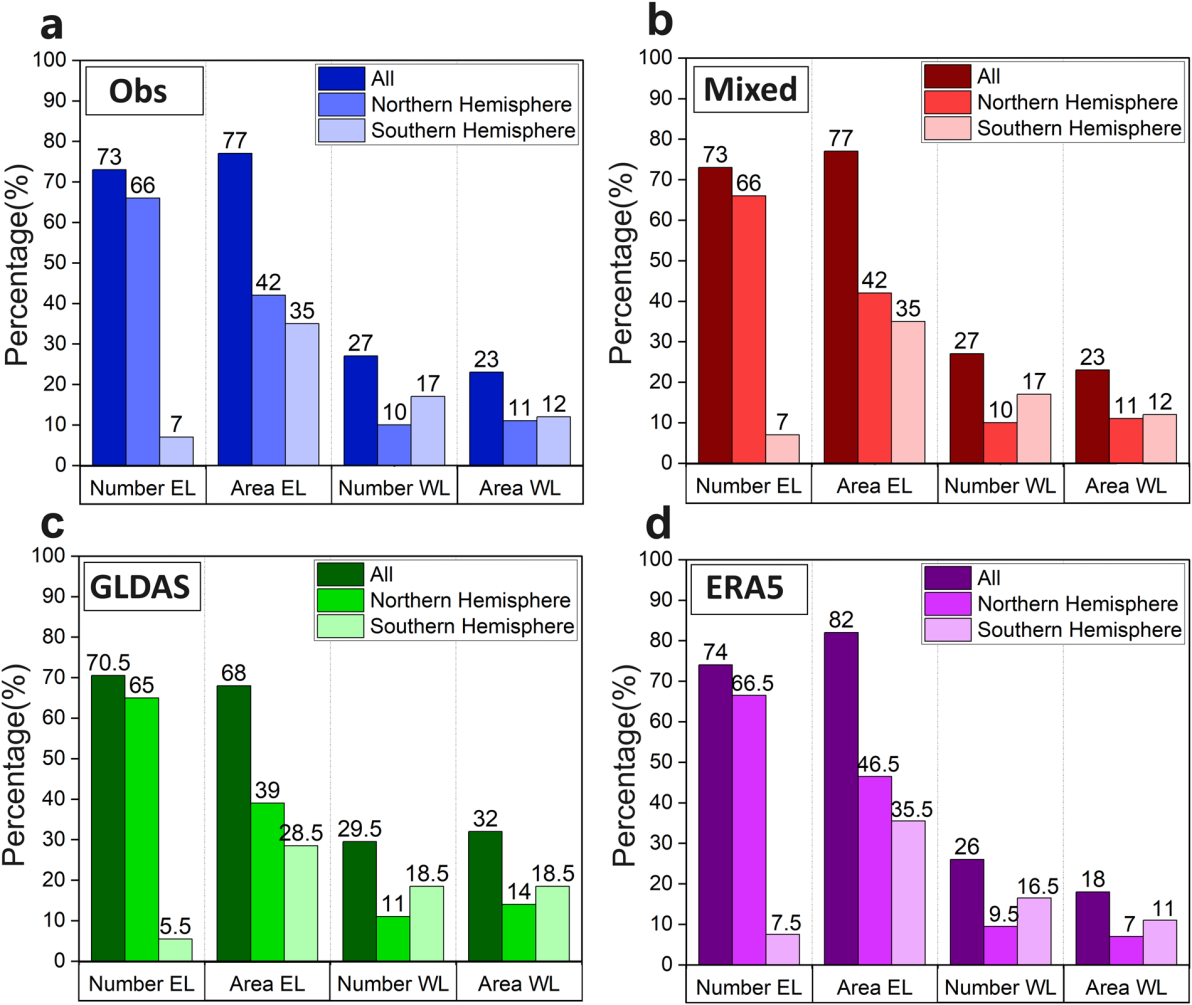
Distributions of water limited (WL) and energy limited (EL) catchments in the Global Hydro-Climatic Data (GHCD) synthesis. Percentages of WL and EL catchment numbers and areas, globally and in the Northern and Southern Hemisphere based on dataset (Table 1): (**a**) “Obs”, (**b**) “Mixed”, (**c**) “GLDAS”, and (**d**) “ERA5”.

The selected non-overlapping catchments represent a wide range of different catchment-wise linked water flow, storage change, and associated water balance, soil moisture and air temperature conditions that jointly prevail and co-vary in various hydrological catchments around the global land area. As such, they also represent different water or energy limited conditions for the catchment characteristic long-term average ratio of actual evapotranspiration to precipitation (ET/P) and its relationship with the long-term average aridity index (PET/P); PET is the long-term average potential evapotranspiration, calculated based on Langbein^33^ for each catchment in GHCD. A catchment is classified as water limited (WL) if PET/P > 1 and as energy limited (EL) if PET/P < 1, with the WL and EL catchments differing in their characteristic ET/P and R/P relationships to PET/P, which are also linked by catchment-wise water balance more or less as R/P≈1-ET/P. The implications of different datasets for the WL and EL classification of catchments are thus relevant for the dataset-comparative research questions (i-iii) that GHCD should contribute to answering. Figure 3 shows the distributions of WL and EL classified catchments around the world, and in the Northern and the Southern Hemisphere considering the asymmetric latitude ranges and associated hydro-climatic conditions of catchments in the two hemispheres. The dataset implications are largely consistent in terms of the WL and EL catchment classification (Fig. 3): the EL catchment area is overall greater than the WL catchment area, globally and in both hemispheres; the number of EL catchments is also mostly greater than that of WL catchments, except in the Southern Hemisphere, which has more (but still with a smaller total area of) WL than EL catchments. The overall dataset agreement seen in Fig. 3 implies a robust EL and WL classification, which thus should not propagate any considerable PET/P based classification uncertainties further to the ET/P and R/P relationship implications of the different datasets.

### Aggregation and averaging of gridded data over each catchment

While the measured stream discharge data in GSIM directly determine the 1561 GHCD catchments and their catchment-average R in “Obs” and “Mixed”, and ET in “Obs”, the other variable data in the comparative datasets are obtained from global gridded products. To provide consistent catchment-average data for the latter, we first extracted the gridded data within the boundary (i.e., the water divide) of each catchment and further spatially interpolated these to produce the spatially aggregated and averaged data for each catchment. Variable values in grid cells that intersect (fully or partially) with a catchment boundary were selected and included in the catchment-wise data aggregation through area-weighted averaging, with the boundary cell weights determined by the intersection area relation to total catchment area. This was done for the finest temporal resolution data (mostly monthly) for the gridded variables, yielding a single catchment-average time series with this resolution (and further a corresponding catchment-average time series with the coarser annual resolution, and an associated long-term average value) for each variable in each catchment.

For comparison with the total average storage change (DS) given by water balance closure for each catchment, we also separately calculated the associated SM change component (DSM; in fraction units per year corresponding to those of relative area-normalized SM, e.g., mm/mm). This was calculated for each catchment based on average SM over a moving 3-year window, with the SM change in time (DSM) quantified from each 3-year window to the next. The 3-year window was chosen to facilitate a balanced capture of both inter-annual variations and longer-term trends in the soil moisture storage change (DSM) over the total 30-year data period, suitable for the targeted comparison with such variations and trends in total average storage change (DS).

The comparison between DS and DSM is interesting and important as an independent test of relevance and realism in the DS implications of the different datasets, as SM and its changes extend down to and are hydraulically linked with the groundwater table and associated groundwater storage change^34^. With the overwhelmingly largest storage of liquid freshwater on Earth^35^ being groundwater, the groundwater storage changes (like the SM storage changes) prevail beneath the entire area of each catchment (and any land area). As such, the groundwater storage changes commonly dominate the total water storage changes (DS), as the entire land area with groundwater storage is generally much larger than the land area covered by the liquid surface water of lakes, wetlands, and rivers/streams. In polar and other cold regions with glaciers and/or permafrost, changes in frozen water storage also contribute to total DS. Outside of the cold areas with glaciers and continuous permafrost, however, the common dominance of groundwater relative to surface water storage changes in the DS of liquid water, and the close subsurface relationship of groundwater storage change with DSM imply that the independent data for DSM in each dataset can be expected to reflect and be consistent with at least the directional sign of total DS (positive/negative for storage increase/decrease). As such, the DSM data can be used as a check of internal data consistency with the DS direction implied by each dataset.

**Fig. 4:**
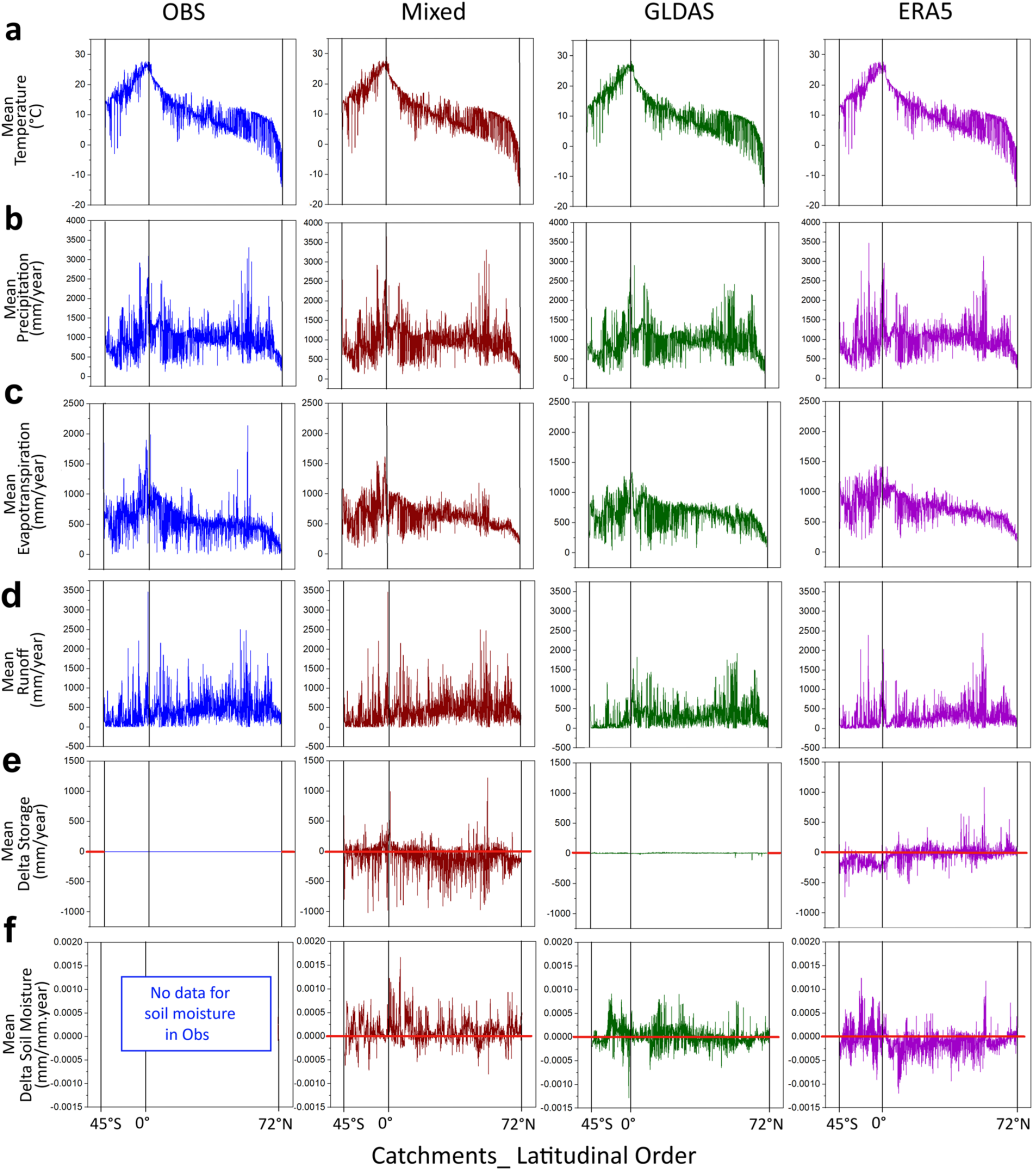
Latitudinal variability of long-term catchment-average air temperature, water fluxes, and water storage changes. Results are shown for all comparative datasets (columns of panels) and all catchments in latitudinal order (45°S-72°N), in terms of the catchment-wise linked long-term (1980–2010) average values of: (**a**) air temperature, (**b**) precipitation, (**c**) evapotranspiration, (**d**) runoff, (**e**) storage change, and (**f**) soil moisture change. Each column of panels shows results for one of the datasets (Table 1); in these and all following figures comparing the datasets, a specific color is used for each dataset: blue for “Obs”, red for “Mixed”, green for “GLDAS”, purple for ERA5”. The “Obs” dataset assumes negligible storage change, as seen in the associated zero change line in panel (**e**), and does not include any own data for soil moisture in panel (**f**). The red lines in panels (**e**) for storage change and (**f**) for soil moisture change show the zero change levels for these variables.

**Fig. 5:**
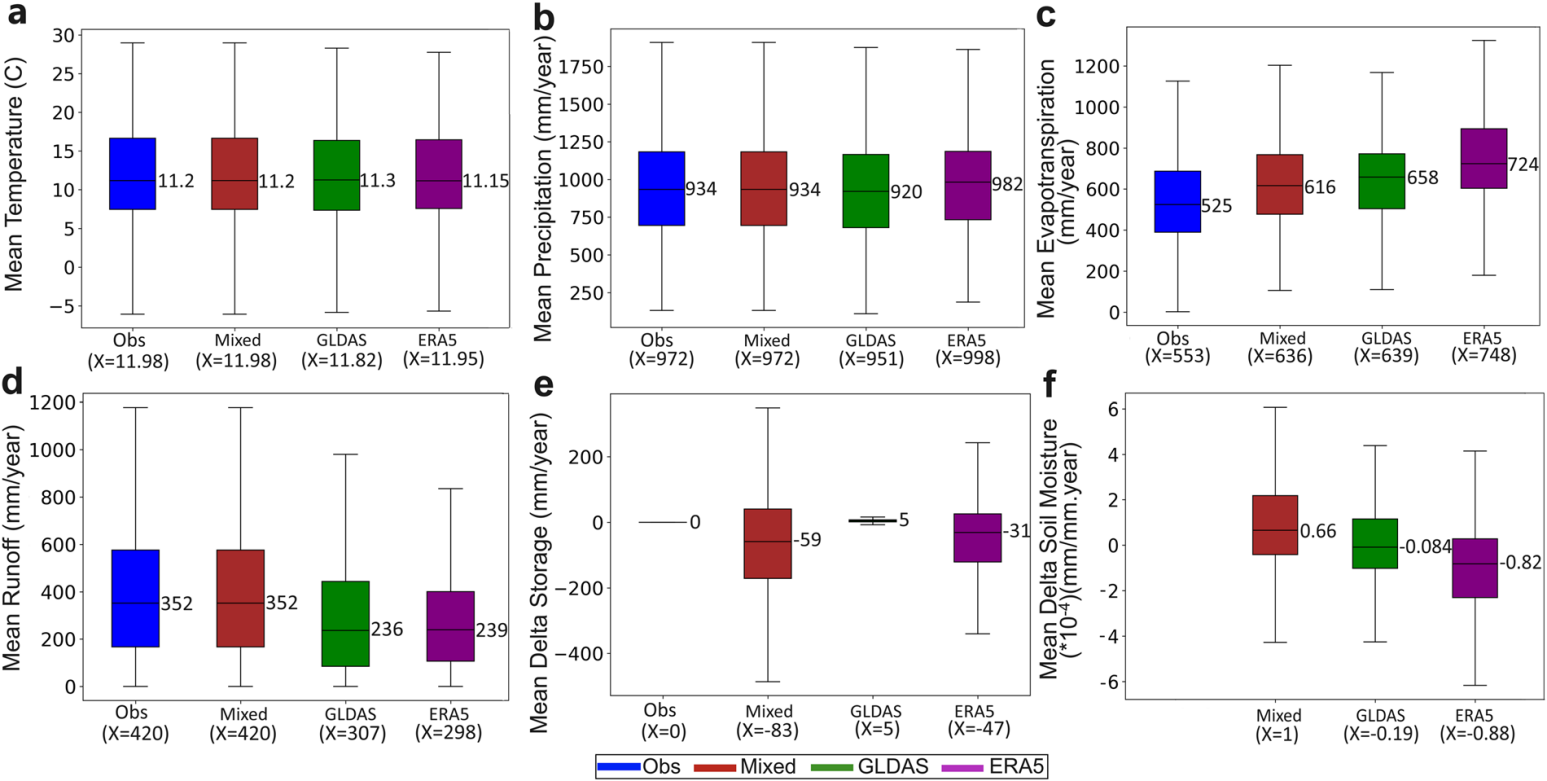
Boxplot statistics of long-term catchment-average variable values. The statistics regard all individual catchments and associated dataset results seen in Fig. 4, and are shown here for the catchment-wise linked long-term average variables of: (**a**) air temperature, (**b**) precipitation, (**c**) evapotranspiration, (**d**) runoff, (**e**) storage change, and (**f**) soil moisture change. All panels compare results for the different datasets (Table 1). The “Obs” dataset assumes negligible storage change, as seen in panel (**e**), and lacks data for soil moisture and is therefore not included in panel (**f**). Each boxplot shows the median value (mid-line number), interquartile range (IQR: box, showing the middle 50% of the data), and data range up to 1.5 times below the 25% and above the 75% quartile values of the IQR; the X values given below the plots show the arithmetic mean data values for each dataset.

Figure 4 shows the latitudinal distributions of the long-term average water fluxes (P, ET, R) and the long-term average storage changes (DS) these imply, along with the associated SM changes (DSM), based on the data in the different comparative datasets. Figure 5 further shows the corresponding statistics of these long-term average values across all catchments for each dataset.

## Data Records

The data from all comparative datasets are included in the completely new synthesis of Global Hydro-Climatic Data (GHCD), available with open access from the Zenodo repository^32^ (10.5281/zenodo.10932898) as a single rar file that contains five separate folders with the data and information provided in GHCD.

Folder 1 contains the catchment polygon shapefiles, used to extract the associated data from the global datasets and further aggregate them over each catchment to produce the catchment-average data time series. The catchment polygons were obtained from the GSIM data^36,37^ and are renamed according to the catchment names used in the new data synthesis of GHCD. A csv file named ‘Catchment_Info.csv’ in Folder 1 lists the GHCD catchment names, their corresponding name in GSIM, the country in which the catchment outlet (hydrometric station) is located, and the catchment area in km^2^ as reported in GSIM^36,37^.

Folders 2–5 provide catchment-average monthly and annual time series for the variables P, ET, R, DS, SM, and T for the 1561 non-overlapping study catchments and the four comparative “Obs”, “Mixed”, “GLDAS”, and “ERA5” datasets included in GHCD (with only annual time series and long-term average values for ET and DS, and no ground observation for SM, in “Obs”). The catchment-wise data time series are provided as “.csv” files, separately for each variable and catchment. Each Dataset folder contains an (i) Annual folder, (ii) Monthly folder, (iii) “DatasetX_AnnualDataSummary.csv” file, and (iv) “Readme_Data Columns and Variable Units.txt” file.

The “Readme_Data Columns and Variable Units.txt” file provides complete information for the variables included in the Annual and Monthly folders, and the associated data source and origin, variable units, and column names of the time series in the CSV files. For the SM variable, the rootzone depth for the soil moisture profile considered in the associated reanalysis products of the “Mixed” (GLEAM), “ERA5”, and “GLDAS” datasets is also reported for possible further applications. The users of GHCD are recommended to read this file before using the variable time series in the Annual or Monthly folders.

The “DatasetX_AnnualDataSummary.csv” file included in Folders 2–5 provides brief summary information for all data included for the 1561 non-overlapping catchments in all comparative datasets of GHCD. The data summary in this file includes the catchment-wise long-term average values of P, R, ET, DS, SM, DSM, T, and PET; related to the quality checks outlined in the Technical Validation section below, it also includes corresponding values for the relative indices PET/P, ET/P and R/P, and the DS-implied average CWLC for all catchments; moreover, it includes the catchment names, the country, the latitude and longitude coordinates of the catchment outlet locations, and the catchment areas in km^2^.

## Technical Validation

### Quality checks for catchment selection

The GSIM data, based on which the GHCD catchments were selected, include monthly discharge time series at the outlets (hydrometric stations) of 30,958 hydrological catchments around the world, with different time spans of continuous data from the beginning of 19^th^ century up to around 2016^37^. From GSIM, we first selected 6696 catchments based on the criterion of having at least 300 non-missing monthly R values (corresponding to 25 years) within the period 1980–2010.

Quality control of the polygons for these catchments was done by checking the reported catchment area against the calculated area based on the shapefile provided in GSIM, and catchments were excluded if these areas did not match. The catchment polygons were also checked for strangely delineated catchments, which were then also removed; for example, some triangle polygons reported as catchments in GSIM were associated with multiple intersecting polygons in the Geographical Information System (GIS) software. The data for the other variables (P, ET, SM, and T) in each remaining GSIM catchment were further also checked, based on which we removed catchments with not-a-number (NaN) data, or with physically unreasonable variable data values (impossibly high or low, e.g., with long-term average R greater than the corresponding long-term average P, implying negative long-term average ET).

For selection of only non-overlapping catchments, we finally distinguished these catchments from overlapping ones based on intersection area. We calculated the percentage of intersection area within the total area of each remaining (that far not removed) catchment in GSIM, and removed the smaller catchments that shared more than 90% of their total area with a bigger catchment, keeping only the latter as a non-overlapping catchment in the final catchment selection. We further validated this selection by a manual check of catchments using ArcGIS Pro software, leading to the final selection of 1561 non-overlapping catchments (Fig. 1) covering a total land area of ∼36*10^6^ km^2^, i.e., nearly one-third of the global land area (excluding Antarctica).

### Comparative water balance checks

The “GLDAS” dataset shows near-zero average storage change in terms of DS and consistently near-zero average soil moisture change (DSM). This is consistent with the assumption of small average storage change (DS≈0) used to model ET in “Obs” (Fig. 5). The internal “GLDAS” consistency between average DS and average DSM, and the resulting consistency with the “Obs” assumption of average DS≈0 apply similarly over all latitudes, in the warmer catchments between 45° S and 60° N as in the colder boreal and arctic ones north of 60° N (Fig. 4e); this overall consistency indicates only small influences of glacier and/or permafrost changes on the total average DS and DSM in the cold catchments. The “Mixed” and “ERA5” datasets instead show overall average storage depletion, i.e., large negative average DS, continuously over the whole 30-year data period, with median DS values of -59 and -31 mm/year, respectively (Fig. 5). Geographically, however, “ERA5” shows divergent DS directions, with highly negative average DS over the Southern Hemisphere, while average DS is around zero over the whole range of warm to cold catchments in the Northern Hemisphere (purple line, Fig. 4e). The near zero DS implication of “ERA5” for the Northern Hemisphere agrees with that of “GLDAS” and is also internally consistent with average DSM also being around zero for “ERA5” (purple line, Fig. 4f); this Northern Hemisphere consistency also applies across all latitudes, from the equator to above 70° N, thereby indicating only small influences of glacier and permafrost changes on the total average DS in the cold northern and arctic catchments. For the Southern Hemisphere, however, the “ERA5” implications of highly negative average DS while average DSM fluctuates mostly above zero are internally inconsistent; this inconsistency applies across nearly all Southern Hemisphere catchments, from the equator to 45° S, and is not explainable by cold-region glacier and/or permafrost change effects. The “Mixed” dataset further shows analogous inconsistency for the Northern Hemisphere, with a continuous 30-year average storage depletion (negative average DS, red line, Fig. 5e) while average SM instead continuously increases (positive average DSM, red line, Fig. 5f); also in this case, there is no indication of potential explanatory cold-region glacier and/or permafrost change effects.

**Fig. 6:**
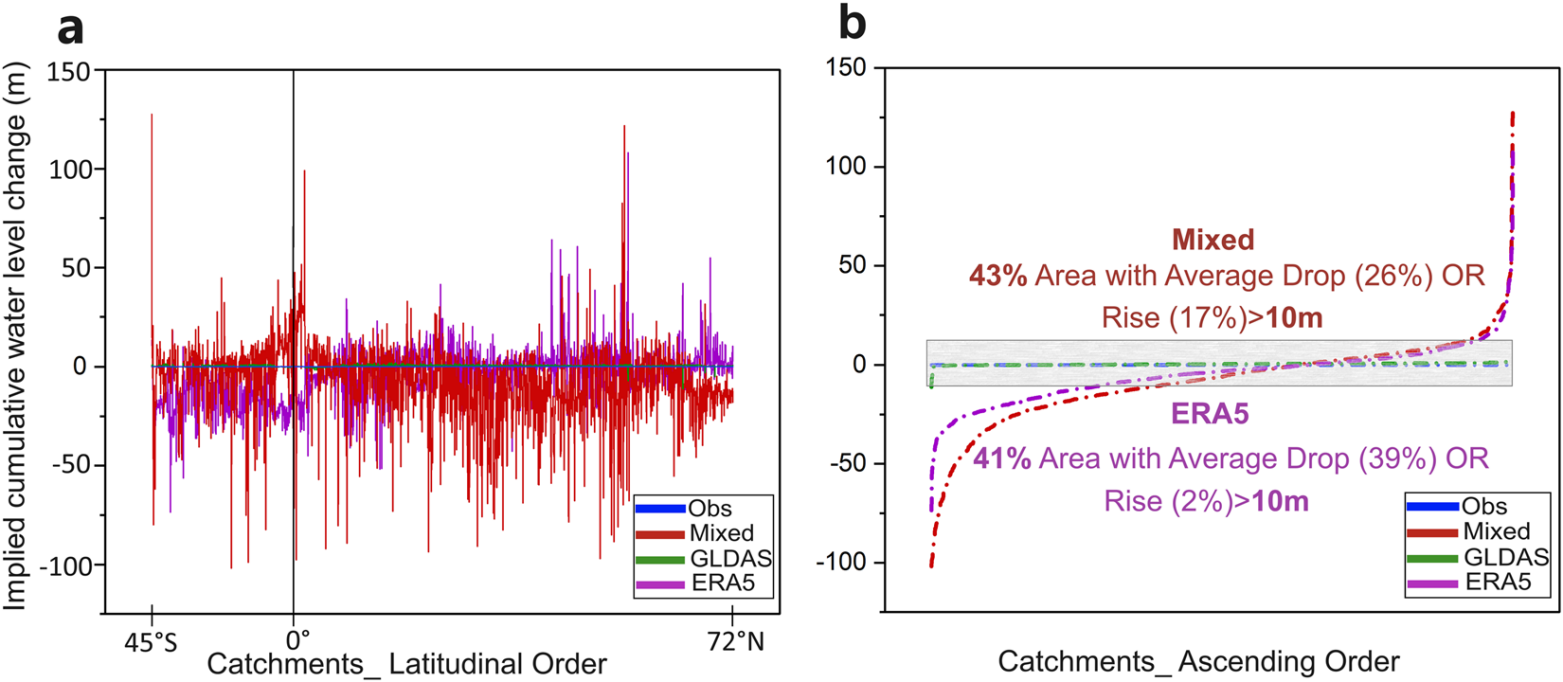
Data implications for cumulative water level change (CWLC). CWLC values implied by the different datasets are shown here for the 1561 catchments in: (**a**) latitudinal order, and (**b**) ascending CWLC order. The shaded field in panel (**b**) shows the CWLC value range of ±10 m.

The realism of DS implications is further checked by the associated indications for catchment-average cumulative water level change (CWLC) over the whole 30-year data period (Fig. 6). Based on the average DS rate (mm/year) obtained from each dataset, a corresponding average CWLC is calculated as the product of average DS and an average porosity example of 0.3 for the geological formations that contain the groundwater beneath the entire land surface area of each catchment (Fig. 6). For the “Mixed” and “ERA5” datasets, the CWLC values emerge as clearly unrealistic catchment-average groundwater level drops or rises by ±100 m and ±50 m, respectively, for some catchments (Fig. 6a; ±50 m rise is also mostly physically impossible without flooding the whole catchment). “Mixed” and “ERA5” also show unrealistic average CWLC across the catchments, with more than ±10 m rise or drop over more than 40% of the total land area covered by the catchments (Fig. 6b). In contrast, the “GLDAS” dataset implies (consistently with what the “Obs” dataset assumes) much smaller and more realistic average CWCL, well below ±10 m (grey field in Fig. 6b) and near zero across most catchments.

**Fig. 7:**
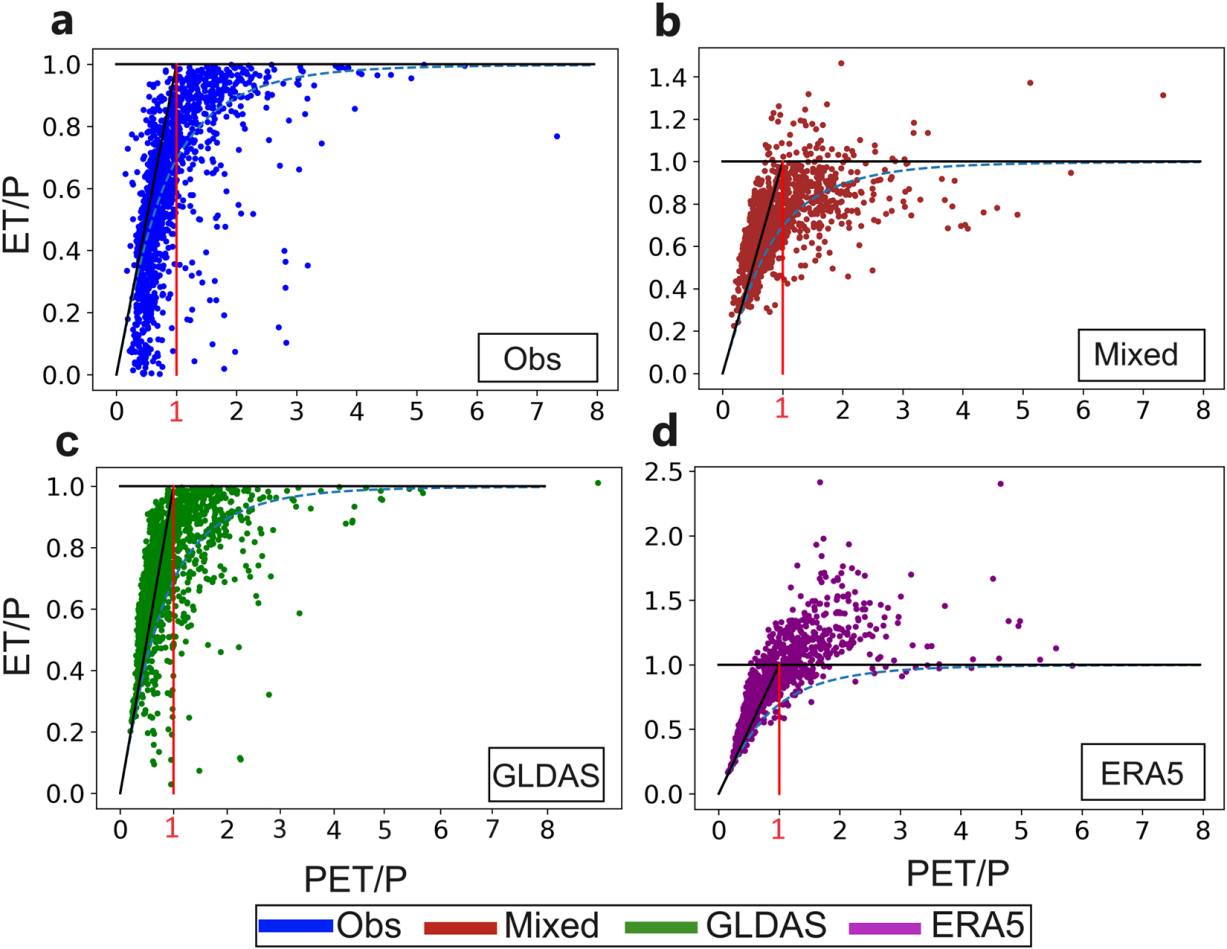
Precipitation (P) normalized actual (ET) and potential (PET) evapotranspiration in Budyko space. Data are shown for all catchments based on the comparative datasets: (**a**) “Obs”, (**b**) “Mixed”, (**c**) “GLDAS”, and (**d**) “ERA5”. The black line for ET/P = PET/P is the theoretical upper ET/P limit if PET/P ≤ 1, under which conditions catchments are classified as energy limited (Fig. 3). The black horizontal line of ET/P = 1 is the theoretical upper ET/P limit if PET/P ≥ 1, under which conditions catchments are classified as water-limited (Fig. 3).

For further check of realism, Fig. 7 illustrates the different dataset implications for the ET/P relationship with PET/P, plotted in Budyko space^33,38^. This is a widely used approach for characterising terrestrial water conditions^39–42^ and the overall dataset consistency seen in Fig. 3 implies a robust PET/P based classification of EL and WL catchments. Dataset differences in this classification thus do not underlie the major ET/P relationship differences seen for the comparative datasets in Fig. 7. Specifically, the “Mixed” and “ERA5” datasets yield average ET/P that for many catchments is considerably greater than the theoretical upper Budyko limit of long-term average ET/P ≤ 1. This reflects an unrealistic water balance closure (rather than divergent PET/P based classification of EL or WL catchments) by the “Mixed” and “ERA5” data, in that a considerable amount of extra water, beyond that provided by P minus the part going to feed R, is needed to feed the large average ET implied by these datasets. This extra water must then come from continuous water storage depletion (negative average DS), which indeed is also what these datasets imply on average across all catchments (Figs 4, 5).

The “Obs” and “Mixed” datasets include the same R, P and T data, and differ only in their modelled ET data, with this ET modeling difference thus leading to the unrealistic DS and CWLC implications of “Mixed”. The “Mixed”, “GLDAS”, and “ERA5” datasets include different data-model data combinations, which are clearly consistent for the climatic T and P variables (Fig. 5a-b) but differ considerably for the landscape water fluxes ET and R calculated by the different types of global modelling underlying these datasets (Fig. 5c-d). On the one hand, the land surface modeling of GLDAS clearly considers and focuses on representing well the landscape water balance of the input water flux P with both the vertical ET and the lateral R fluxes in the landscape (green bar, Fig. 5e). On the other hand, the global ET-targeting GLEAM in “Mixed” and overall climate-targeting ERA5 modelling mainly focus on representing well the vertical land-atmosphere coupling by the ET fluxes; for ET in “ERA5”, we also clarify that GHCD includes data for the “Total Evaporation” variable, which aggregates all vertical flux components from land to the atmosphere: soil evaporation, vegetation transpiration, interception loss, and open water evaporation. With their main focus on the vertical land-atmosphere coupling, it is not a primary target of the GLEAM and ERA5 modelling to represent well the lateral water flows of R and related landscape water balance (red and purple bars, Fig. 5). However, these global datasets are still widely used also for landscape water purposes, and the users need to know the performances and understand the implications of these datasets for the landscape water system in different parts of the world; the inclusion of “Mixed” and “ERA5” in the GHCD synthesis facilitates this.

Furthermore, even though the combined water balance and DS implications of “Mixed” and “ERA5” emerge as unrealistic for some regions and on average over the world, parts of these datasets may still be sufficiently relevant for at least some of the water fluxes (P, ET, R) and/or SM in some catchments and parts of the world. GHCD therefore provides the water flux, SM and T data, along with the DS and other implication measures for all included datasets. This gives GHCD users access to all catchment-wise linked water and hydro-climatic data so that they can compare, check and determine if, to what degree, and for which variables and implications the data in for different variables in various datasets are sufficiently realistic and relevant to use, or require further testing against independent data for the specific catchment(s), region(s), and investigation scale(s) of interest. The water balance checks and implication measures provided for all comparative datasets in GHCD can further be tested by comparison with independent local measurements and satellite data that can be used to quantify catchment-average DS, CWCL and/or ET/P^27–29^.

### Dataset agreement checks

As part of the quality check, there is also a need to identify possible important dataset agreements. Such agreements are valuable, as they indicate relatively robust knowledge, with relatively small uncertainties, for the related water aspects and conditions. One such important agreement is that the four datasets yield consistent long-term average values and spatial variations of T and P, i.e., the atmospheric driver variables (Figs. 4a,b and 5a,b).

**Fig. 8:**
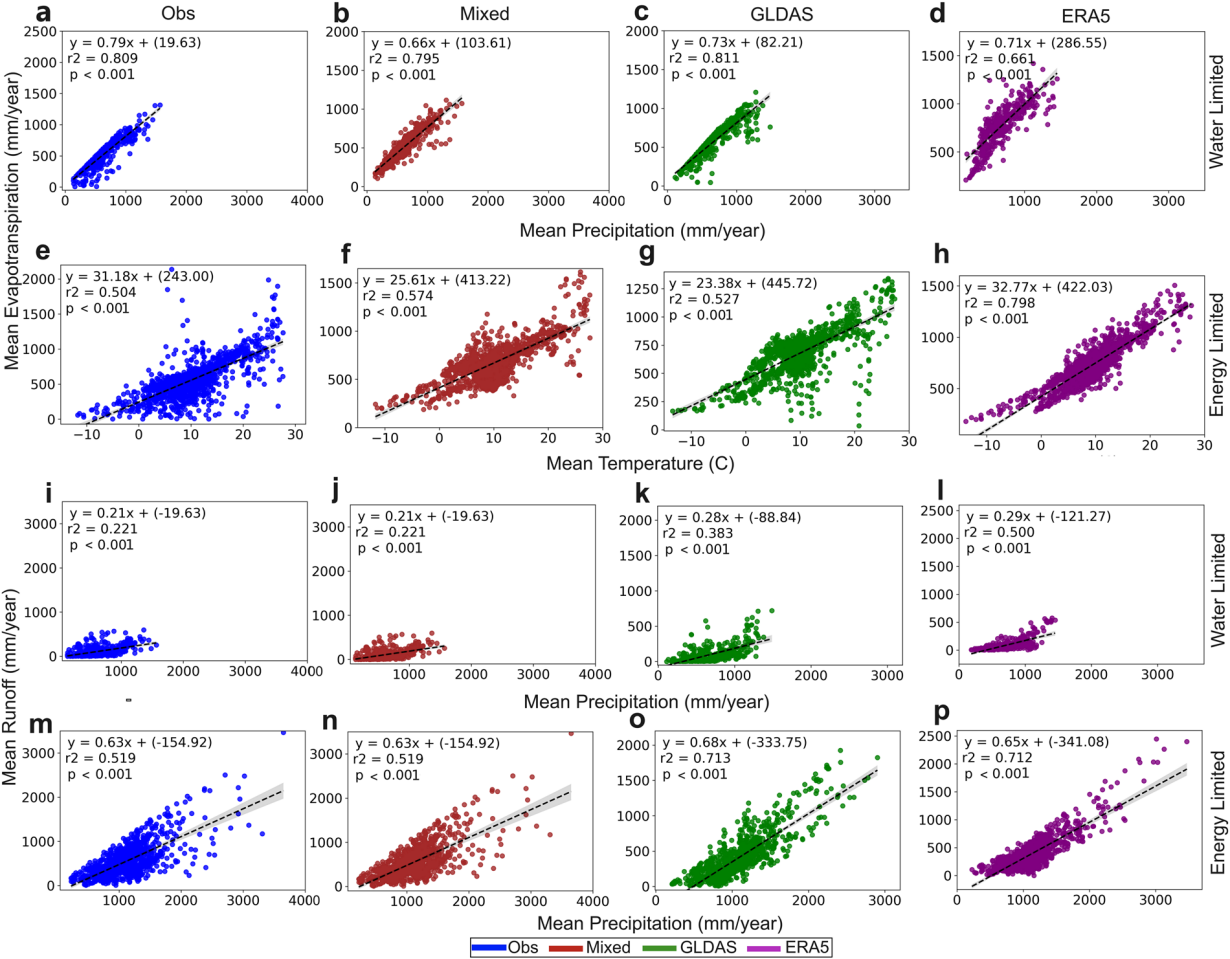
The strongest ET and R correlations with either P or T. Data are shown for evapotranspiration (ET) versus precipitation (P) for: (**a**–**d**) water limited (WL) catchments, and (**e**–**h**) energy limited (EL) catchments; and for runoff (R) versus P data for: (**i**–**l**) WL catchments, and (m-p) EL catchments. Each panel column shows results for a specific dataset: “Obs”, “Mixed”, “GLDAS” or “ERA5”. The shaded area around the regression lines shows the 95% confidence interval for each line, and the r^2^ and p values quantify the coefficient of determination and the statistical significance level of each line fit line, respectively; the p level is consistently low, p < 0.001, implying significant linear correlation at this level.

Moreover, all datasets agree on the overall correlation patterns of the spatial variations of long-term average ET and R with those of the atmospheric driver variables P and T (Fig. 8). Overall, the ET and R variations emerge as more strongly correlated with the P than the T variations. This is illustrated in Fig. 8, with the data for average ET and R plotted against the associated P or T data, depending on which is most strongly correlated with ET and R, consistently for all datasets, in water limited (WL) and energy limited (EL) catchment conditions. Only in the EL catchments do the ET variations emerge as relatively well correlated with T (Fig. 8e–h), but ET is also relatively well correlated with P in these catchments (with coefficient of determination, r^2^, values in the range 0.3–0.6; not shown). ET is also more strongly correlated with P in the WL catchments (Fig. 8a–d) than it is with T in the EL catchments (Fig. 8e–h). In the EL catchments, R is more or less equally well correlated with P (Fig. 8m–p) as ET is with T in these catchments, while R emerges as relatively weakly correlated with P in the WL catchments (Fig. 8i–l) but still more strongly so than with T (with which R exhibits no correlation at all, not shown).

Another important dataset agreement is that the “Mixed”, “GLDAS” and “ERA5” datasets all show that average DS is overall relatively small (within ±5%) compared to average ET (Fig. 5). The “Obs” dataset uses the assumption of average DS≈0 to model average ET, and the other datasets thus largely support this simplifying assumption. Furthermore, the implications for average DS are similar for each dataset (even though they differ between datasets) over all Northern Hemisphere latitudes, from the warm catchments by the equator to the cold boreal and arctic catchments north of 60° N (Fig. 4e). This latitudinal consistency for each dataset implies that the datasets agree on small glacier and/or permafrost change influences on average DS; potential considerable such cryosphere change influences could otherwise have contributed to explain the DS disagreements between datasets

## Usage Notes

For use of the GHCD synthesis with its four comparative datasets we have in the Technical Validation section identified some key dataset agreements and disagreements. For the disagreements we have also checked the divergent dataset representations for possible biases, realism, and internal cross-variable consistency. These checks have mostly regarded average conditions over all (or distinct large groups of) catchments, in order to identify bias and consistency tendencies at large continental-global scale for the different datasets. The identified divergent dataset tendencies different GHCD variables need to be considered in large-scale multi-catchment studies, e.g., for determining which dataset(s) to mostly rely on and use in such studies.

The types of checks included in the Technical Validation section also provide guidance for dataset choices and relevant aspects to check in smaller-scale studies of individual or few catchments. For such studies, the checks in the Technical Validation section also show which types of agreements and disagreements can on average be expected among the different datasets, as a basis for identifying possible divergent results in the own study catchments. The research and understanding of water conditions on land can be significantly advanced by deciphering the reasons for such findings that may diverge from average dataset agreement/disagreement expectations.

With regard to average dataset agreements, the GHCD users should note that all datasets largely agree on the long-term average values and spatial variations of the atmospheric driver variables T and P, and on how the spatial variations of the landscape water fluxes ET and R, and the related water storage changes DS, correlate with these. The datasets also agree on that DS is on average relatively small, within ±5%, compared to average ET.

With regard to average dataset disagreements, the GHCD users should note that the “Mixed” and “ERA5” datasets emerge as rather unrealistic in their representation of long-term average ET, which for many catchments is represented as considerably greater than the total long-term average P input of water. As a consequence, these datasets then also show, on average, systematic continuous water storage depletion (negative DS) over the whole 30-year data period. The DS directions in the individual catchments are further, on average, inconsistent with those in the same datasets for the corresponding soil moisture change (DSM), which is an integral part of total DS.

The GHCD users should also note that the comparative datasets are not and do not need to be fully independent. Rather, the similarities combined with some clear distinction help identify some main reasons for the result differences between datasets. For example, the “Obs” and “Mixed” datasets share the same R, P and T data, and the comparison between them clearly indicates their different modeling of ET as the reason for their divergence in average DS. Further, the global GLDAS and ERA5 modelling may include similar atmospheric forcing representations, yet these two datasets yield distinctly different modelled ET and resulting DS. Instead, the inclusion of GLEAM for the modelled ET in “Mixed” yields global average DS similar to that of “ERA5”; this points at their analogous targeting of the vertical ET fluxes, without much focus on the lateral R fluxes and resulting water balance in the landscape, as a possible reason for their similar DS implications of continuous 30-year average water depletion globally. Overall, the datasets in GHCD can facilitate the further research needed to decipher if, to what degree, and why datasets with some similarities and certain clear distinctions agree or disagree in their implications for answering essential open questions (e.g., i-viii in Background & Summary) for the water fluxes, storage changes, and their balances around the global land area.

## Data Availability

The data for this study was processed and analyzed in Python (version 3.9), with cross-checking and corrections of spatial coordinates conducted using ArcGISPro. The codes used for data analysis, producing figures, and finding non-overlapping catchments are available as part of the data record (10.5281/zenodo.10932898)^32^.

## References

[1] Zarei, M. & Destouni, G. Research Gaps and Priorities for Terrestrial Water and Earth System Connections From Catchment to Global Scale. *Earths Future* **12**, e2023EF003792 (2024).

[2] Gudmundsson, L., Seneviratne, S. I. & Zhang, X. Anthropogenic climate change detected in European renewable freshwater resources. *Nat. Clim. Change* **7**, 813–816 (2017).

[3] Gudmundsson, L. *et al*. Globally observed trends in mean and extreme river flow attributed to climate change. *Science* **371**, 1159–1162 (2021). 33707264 10.1126/science.aba3996

[4] Teuling, A. J. *et al*. Climate change, reforestation/afforestation, and urbanization impacts on evapotranspiration and streamflow in Europe. *Hydrology and Earth System Sciences* **23**, 3631–3652 (2019).

[5] Sang, L. *et al*. Effects of agricultural large-and medium-sized reservoirs on hydrologic processes in the arid Shiyang River Basin, northwest China. *Water Resources Research* **59**, e2022WR033519 (2023).

[6] Althoff, D. & Destouni, G. Global patterns in water flux partitioning: Irrigated and rainfed agriculture drives asymmetrical flux to vegetation over runoff. *One Earth* **6**, 1246–1257 (2023).

[7] Gedney, N. *et al*. Detection of a direct carbon dioxide effect in continental river runoff records. *Nature* **439**, 835–838 (2006). 16482155 10.1038/nature04504

[8] Piao, S. *et al*. Changes in climate and land use have a larger direct impact than rising CO2 on global river runoff trends. *Proc. Natl. Acad. Sci.* **104**, 15242–15247 (2007). 17878298 10.1073/pnas.0707213104 PMC1978487

[9] Pan, M. *et al*. Multisource estimation of long-term terrestrial water budget for major global river basins. *J. Clim.* **25**, 3191–3206 (2012).

[10] Miralles, D. G. *et al*. El Niño–La Niña cycle and recent trends in continental evaporation. *Nat. Clim. Change* **4**, 122–126 (2014).

[11] Zhang, Y. *et al*. Multi-decadal trends in global terrestrial evapotranspiration and its components. *Sci. Rep.* **6**, 19124 (2016). 26750505 10.1038/srep19124 PMC4707530

[12] Yang, H., Huntingford, C., Wiltshire, A., Sitch, S. & Mercado, L. Compensatory climate effects link trends in global runoff to rising atmospheric CO2 concentration. *Environ. Res. Lett.* **14**, 124075 (2019).

[13] Bring, A. *et al*. Implications of freshwater flux data from the CMIP5 multimodel output across a set of Northern Hemisphere drainage basins. *Earths Future* **3**, 206–217 (2015).

[14] Ghajarnia, N., Kalantari, Z. & Destouni, G. Data‐driven worldwide quantification of large‐scale hydroclimatic covariation patterns and comparison with Reanalysis and Earth system modeling. *Water Resour. Res.* **57**, e2020WR029377 (2021).

[15] Berghuijs, W. R., Sivapalan, M., Woods, R. A. & Savenije, H. H. Patterns of similarity of seasonal water balances: A window into streamflow variability over a range of time scales. *Water Resour. Res.* **50**, 5638–5661 (2014).

[16] Lehmann, F., Vishwakarma, B. D. & Bamber, J. How well are we able to close the water budget at the global scale? *Hydrol. Earth Syst. Sci.* **26**, 35–54 (2022).

[17] Knoben, W. J., Woods, R. A. & Freer, J. E. A quantitative hydrological climate classification evaluated with independent streamflow data. *Water Resour. Res.* **54**, 5088–5109 (2018).

[18] Berghuijs, W. R., Harrigan, S., Molnar, P., Slater, L. J. & Kirchner, J. W. The relative importance of different flood‐generating mechanisms across Europe. *Water Resour. Res.* **55**, 4582–4593 (2019).

[19] Schneider, U. *et al*. GPCC full data reanalysis version 7.0: Monthly land-surface precipitation from rain gauges built on GTS based and historic data. (2016).

[20] Do, H. X., Gudmundsson, L., Leonard, M. & Westra, S. The Global Streamflow Indices and Metadata Archive - Part 1: Station catalog and Catchment boundary 10.1594/PANGAEA.887477 (2018).

[21] Gudmundsson, L., Do, H. X., Leonard, M. & Westra, S. The Global Streamflow Indices and Metadata Archive (GSIM) - Part 2: Time Series Indices and Homogeneity Assessment 10.1594/PANGAEA.887470 (2018).

[22] Miralles, D. G. *et al*. Global land-surface evaporation estimated from satellite-based observations. *Hydrol. Earth Syst. Sci.* **15**, 453–469 (2011).

[23] Martens, B. *et al*. GLEAM v3: Satellite-based land evaporation and root-zone soil moisture. *Geosci. Model Dev.* **10**, 1903–1925 (2017).

[24] Rodell, M. *et al*. The global land data assimilation system. *Bull. Am. Meteorol. Soc.* **85**, 381–394 (2004).

[25] Beaudoing, H. & Rodell, M. GLDAS Noah Land Surface Model L4 monthly 1.0× 1.0 degree V2. 0, Greenbelt, Maryland, USA, Goddard Earth Sciences Data and Information Services Center (GES DISC)[data set] (2019).

[26] Hersbach, H. *et al*. Complete ERA5 from 1940: Fifth generation of ECMWF atmospheric reanalyses of the global climate. Copernicus Climate Change Service (C3S) Data Store (CDS) (2017).

[27] Bhattarai, N. *et al*. An automated multi-model evapotranspiration mapping framework using remotely sensed and reanalysis data. *Remote Sens. Environ.* **229**, 69–92 (2019).

[28] Moshir Panahi, D., Kalantari, Z., Ghajarnia, N., Seifollahi-Aghmiuni, S. & Destouni, G. Variability and change in the hydro-climate and water resources of Iran over a recent 30-year period. *Sci. Rep.* **10**, 7450 (2020). 32366897 10.1038/s41598-020-64089-y PMC7198531

[29] Saemian, P., Elmi, O., Vishwakarma, B., Tourian, M. & Sneeuw, N. Analyzing the Lake Urmia restoration progress using ground-based and spaceborne observations. *Sci. Total Environ.* **739**, 139857 (2020). 32758937 10.1016/j.scitotenv.2020.139857

[30] Fan, Y. & Van den Dool, H. A global monthly land surface air temperature analysis for 1948–present. *J. Geophys. Res. Atmospheres* **113** (2008).

[31] World Meteorological Organization. WMO guidelines on the calculation of climate normals (2017).

[32] Zarei, M. & Destouni, G. Global Hydro-Climatic Data (GHCD). *Zenodo*10.5281/zenodo.10932898 (2024).

[33] Langbein, W. B. *Annual Runoff in the United States*. (United States Department of the Interior, Geological Survey, 1949).

[34] Destouni, G. & Verrot, L. Screening long-term variability and change of soil moisture in a changing climate. *J. Hydrol.* **516**, 131–139 (2014).

[35] Oki, T. & Kanae, S. Global Hydrological Cycles and World Water Resources. *Science* **313**, 1068–1072 (2006). 16931749 10.1126/science.1128845

[36] Do, H. X., Gudmundsson, L., Leonard, M. & Westra, S. The Global Streamflow Indices and Metadata Archive (GSIM)–Part 1: The production of a daily streamflow archive and metadata. *Earth Syst. Sci. Data* **10**, 765–785 (2018).

[37] Gudmundsson, L., Do, H. X., Leonard, M. & Westra, S. The Global Streamflow Indices and Metadata Archive (GSIM)–Part 2: Quality control, time-series indices and homogeneity assessment. *Earth Syst. Sci. Data* **10**, 787–804 (2018).

[38] Koppa, A., Alam, S., Miralles, D. G. & Gebremichael, M. Budyko‐based long‐term water and energy balance closure in global watersheds from earth observations. *Water Resour. Res.* **57**, e2020WR028658 (2021). 34219820 10.1029/2020WR028658 PMC8244049

[39] Berghuijs, W., Woods, R. & Hrachowitz, M. A precipitation shift from snow towards rain leads to a decrease in streamflow. *Nat. Clim. Change* **4**, 583–586 (2014).

[40] Roderick, M. L. & Farquhar, G. D. A simple framework for relating variations in runoff to variations in climatic conditions and catchment properties. *Water Resour. Res*. **47** (2011).

[41] Wang, C., Wang, S., Fu, B. & Zhang, L. Advances in hydrological modelling with the Budyko framework: A review. *Prog. Phys. Geogr.* **40**, 409–430 (2016).

[42] Xu, X., Liu, W., Scanlon, B. R., Zhang, L. & Pan, M. Local and global factors controlling water‐energy balances within the Budyko framework. *Geophys. Res. Lett.* **40**, 6123–6129 (2013).

